# Understanding the variability in plasma biomarker levels in the context of established biomarkers of Alzheimer’s disease

**DOI:** 10.1002/alz.090702

**Published:** 2025-01-09

**Authors:** Marina Bluma, Konstantinos Chiotis, Marco Bucci, Irina Savitcheva, Anna Matton, Miia Kivipelto, Andreas Jeromin, Giovanni De Santis, Guglielmo Di Molfetta, Nicholas J. Ashton, Kaj Blennow, Henrik Zetterberg, Agneta K Nordberg

**Affiliations:** ^1^ Division of Clinical Geriatrics, Center for Alzheimer Research, Department of Neurobiology, Care Sciences and Society, Karolinska Institutet, Stockholm Sweden; ^2^ Department of Neurology, Karolinska University Hospital, Stockholm Sweden; ^3^ Theme Inflammation and Aging, Karolinska University Hospital, Stockholm Sweden; ^4^ Medical Radiation Physics and Nuclear Medicine, Section for Nuclear Medicine, Karolinska University Hospital, Stockholm Sweden; ^5^ ALZpath, Inc., Carlsbad, CA USA; ^6^ Department of Psychiatry and Neurochemistry, Institute of Neuroscience and Physiology, The Sahlgrenska Academy, University of Gothenburg, Mölndal, Gothenburg Sweden; ^7^ Institute of Neuroscience and Physiology, University of Gothenburg, Mölndal Sweden; ^8^ Department of Psychiatry and Neurochemistry, Institute of Neuroscience and Physiology, the Sahlgrenska Academy, University of Gothenburg, Mölndal, Gothenburg Sweden; ^9^ Department of Psychiatry and Neurochemistry, Institute of Neuroscience and Physiology, The Sahlgrenska Academy, University of Gothenburg, Mölndal Sweden

## Abstract

**Background:**

Plasma biomarkers are on the verge of being introduced as screening tools for detecting Alzheimer's disease (AD). Yet, previous studies yielded contradictory results regarding the extent to which distinct plasma biomarkers reflect the key pathological changes associated with AD. This study aims to investigate the specific relationship between core AD biomarkers, such as amyloid PET and CSF pTau, and the novel plasma biomarkers.

**Method:**

We analyzed plasma pTau181, pTau217, pTau231, GFAP, NfL, CSF pTau181, Aβ‐PET scans, and MRI/CT visual reads of atrophy (MTA and GCA) in a cohort of memory clinic patients (N=138) who underwent comprehensive clinical assessments at the Memory Clinic, Karolinska University Hospital, Stockholm, Sweden. Patients were classified according to the ATN system, for the presence of Aβ‐deposition (A), tau aggregation (T), and neurodegeneration (N) based on Aβ‐PET, CSF pTau and MRI/CT measurements, respectively. We utilized methods based on multiple linear regression to evaluate the distinct contributions of Aβ‐PET, CSF pTau, and neurodegeneration, on plasma biomarker levels, while also considering demographic variables.

**Result:**

We found significant (p<0.05) positive associations between Aβ‐PET and plasma pTau181, pTau217, pTau231, GFAP, but not NfL. Similarly, significant positive associations were observed between CSF pTau and plasma pTau isoforms and GFAP. After accounting for the effect of Aβ‐PET, only plasma pTau217 and pTau231 remained significantly associated with CSF pTau. Among all, plasma pTau217 and GFAP exhibited the greatest effect sizes to distinguish between A+/A‐, whereas plasma pTau231 and pTau217 for distinguishing T+/T‐. Plasma NfL showed the highest effect size for distinguishing N+/N‐ among all plasma biomarkers. Dominance analysis indicated that Aβ‐PET had the highest relative importance in predicting pTau isoforms and GFAP values, followed by CSF pTau. Notably, pathology‐related AD biomarkers (Aβ‐PET and CSF pTau) explained the largest portion of variance in plasma pTau217(56%), GFAP (24%), pTau181(16.2%), pTau231(17%), but not NfL(0.6%).

**Conclusion:**

Amyloid burden primarily drove changes in levels of the tested plasma biomarkers, except for NfL where atrophy played a role. Unlike plasma GFAP and pTau181, where the effect of CSF pTau was attenuated by the effect of Aβ‐PET, both pTau231 and pTau217 reflected changes in CSF pTau levels. Variance in plasma pTau217 levels appeared to be well explained by AD‐related pathological changes.

